# A multi-center, open label, single group, observational clinical trial to investigate the effects of training on the administration of Cardioplexol™

**DOI:** 10.3389/fcvm.2025.1588088

**Published:** 2025-05-26

**Authors:** Hendrik T. Tevaearai Stahel, Niuscha Taheri, Andreas Winkler, Johannes Hohlfeld, Wolfgang Dietl, Christoph Starck, Arnaud Van Linden, Jan Bidovec, Aaron Imhof, Thierry P. Carrel, Bernard Voet, Thomas Walther, Rainald Seitelberger, Michael Grimm, Christoph Holzinger, Martin Grabenwöger

**Affiliations:** ^1^ Department of Cardiovascular, Inselspital, Berne University Hospital, University of Berne, Berne, Switzerland; ^2^ Department of Cardiovascular Surgery, Clinic Floridsdorf, Vienna, Austria; ^3^ Clinic for Cardiovascular and Endovascular Surgery, University Clinic Salzburg, Salzburg, Austria; ^4^ Clinic for Cardiac Surgery, University Clinic Innsbruck, Innsbruck, Austria; ^5^ Clinic for Cardiac Surgery, University Clinic St. Pölten, St. Pölten, Austria; ^6^ Clinic for Cardiac, Thoracic and Vascular Surgery, German Heart Center Berlin, Berlin, Germany; ^7^ Clinic for Thoracic, Cardiac and Thoracic Vascular Surgery, University Clinic Frankfurt, Frankfurt, Germany; ^8^ Clinic for Cardiac Surgery, University Hospital Zürich, Zürich, Switzerland; ^9^ Voet Consulting, Berlin, Germany; ^10^ Karl Landsteiner Institute of Cardiovascular Research, Vienna, Austria; ^11^ Sigmund Freud Private University, Medical Faculty, Vienna, Austria

**Keywords:** cardiac surgery, cardioplegic solution, cardioplegia, myocardial protection, extracorporeal circulation

## Abstract

**Introduction:**

Cardioplexol™ was recently proven effective and non-inferior to Buckberg's solution in a pivotal Phase-3 clinical trial. We hypothesized here that a standardized training program for surgeons without prior experience of Cardioplexol™ could increase its administration reliability and participate to its overall benefit.

**Methods:**

Open label, single group, observational study involving 29 surgeons from 7 centers in 3 countries. The training program included a theoretical part, and two surgical procedures performed under trainer supervision. In a subsequent evaluation part, surgeons operated on 4 additional patients. The number of major deviations from the pre-defined administration protocol (incorrect volume of initial/second/third/fourth dose, incorrect duration of injection of initial dose, incorrect timing of application of initial/second/third/fourth dose) was set as primary endpoint.

**Results:**

A total of 171 patients were screened of which 157 were operated on (57 in the training part and 100 in the evaluation part). No major deviations were observed. Other outcomes, including postoperative TnT and CK-MB profiles, cumulative inotropic support provided during the first 24 h after myocardial reperfusion, cardiac conversion rate, ICU length of stay, were all similar to or better than the results observed in the previous pivotal study.

**Conclusion:**

Cardiac surgeons not familiar to Cardioplexol™ benefit from a structured and supervised training. This kind of training contributes to improve the efficiency and safety of a new cardioplegic solution such as Cardioplexol™.

**Trial registration:**

[ClinicalTrials.gov]: identifier [NCT03823521, and EudraCT No: 2018-002311-10].

## Introduction

Introduction of cardioplegia some seven decades ago led to extraordinary developments in open-heart surgery, notably through major improvements in operating conditions. Above all, safety in cardiac surgery has been dramatically increased, by attenuating the effects of myocardial ischemia linked to the absence of coronary perfusion during the aortic clamping period. Today, the crystalloid solutions of St-Thomas and Bretschneider, as well as the Buckberg blood cardioplegia introduced more than 50 years ago, are still considered standard options (1–4). Interestingly, although many variants of these solutions, as well as a few new formulations, are in widespread use, the vast majority are still not officially approved as drugs by the registration authorities, and only very few demonstrate high levels of evidence (5).

Cardioplexol™ is a new crystalloid cardioplegic solution for intracoronary use (6–9). It contains potassium chloride, magnesium sulphate heptahydrate, xylitol, and procaine hydrochloride. Cardioplexol™ was recently evaluated in a pivotal phase 3 clinical trial which demonstrated its efficacy and non-inferiority compared with Buckberg's standard solution (10). The analysis of clinical and laboratory parameters also confirmed its safety. However, this study also indicated that surgeons using Cardioplexol™ for the first time, may benefit from a dedicated training program, aimed at minimizing the risk of administration errors and thus contributing to reducing the risk of potentially serious clinical consequences.

Indeed, even though the administration of Cardioplexol™ is straightforward, it differs significantly from usual cardioplegia applications in most clinics. In the first phase 3 clinical trial, a substantial number of administration errors was observed in that the volume and, especially, the timing of initial and subsequent doses of Cardioplexol™ were not strictly in line with the study protocol (10).

The purpose of the present study was therefore to assess the potential of a dedicated controlled training protocol on the rate of correct administration of Cardioplexol™ solution.

## Patients and methods

### Study design

Multi-center, open label, single group, observational study designed to evaluate the effects of a Cardioplexol™ preparation and administration training program proposed to cardiac surgeons and perfusionists without previous experience in the use of this solution. The study was conducted between November 2018 and October 2021 and involved 7 centers in 3 countries: the Clinic Floridsdorf in Vienna, the German Heart Center in Berlin, and the University Hospitals in St. Pölten, Salzburg, Innsbruck, Frankfurt and Zürich.

The study has been conducted within the framework of a national registration procedure for Cardioplexol™ in Switzerland and, in parallel, in a decentralized registration procedure (DCP) in 10 other European countries. The reference member state for the DCP was Austria.

### Ethics approval and consent to participate

The study protocol was approved by the “Ethikkommission der Stadt Wien,” the “Ethikkommission der Universität Frankfurt” and the “Kantonale Ethikkommission, Kanton Zürich” for study site(s) in Austria, Germany and Switzerland respectively, as well as by the Austrian BASG (Bundesamt für Sicherheit im Gesundheitswesen), German BfArM (Bundesinstitut für Arzneimitel und Medizinprodukte) and Swissmedic (Swiss Agencies for Therapeutic Products) regulatory agencies. Each patient included in the study was informed by the investigator at least 24 h prior to the intervention and provided preoperative written informed consent.

### Study population

Patients aged between 18 and 80 years scheduled for a primary elective cardiac coronary artery bypass graft (CABG) and/or a cardiac valve repair/replacement via full or hemi-sternotomy, under cardiac arrest and assistance of a heart lung machine were considered eligible for enrollment in the trial. Exclusion criteria were the same as those defined for the pivotal study (10), i.e., pre-operative LVEF < 30%, IABP, catecholamine support, myocardial infarction within 7 days, previous cardiac surgery including pace-maker or ICD, active myocarditis and/or endocarditis, aortic valve insufficiency (severity grade >1), history of atrial, dialysis or pre-operative creatinine >2.0 mg/dl, anti-vitamin K treatment or known hematologic disorder, HIT, patient is pregnant or lactating, intravenous drug users, alcohol abusers, prisoners, patients institutionalized or unable to give informed consent.

### Training program and evaluation

The objectives of the training program were, on the one hand, to teach how to prepare Cardioplexol™ correctly and, on the other hand, how to administer it correctly. The “preparation” part was mainly, but not exclusively, addressed to cardiotechnicians, while the “administration” part was mainly, but not exclusively, aimed at surgeons.

The study was carried out in two distinct parts. In part 1, the participating surgeons were trained on site according to a standardized training program. The impact of the provided training was then evaluated in part 2:

- Part 1 (Training): Details of the training program are described in Supplementary Material 1. After successful completion of a theoretical part, the surgeon was authorized to perform the first two Cardioplexol™ surgeries under the supervision of a trainer (Author HT, who is a surgeon with extensive theoretical and clinical experience in the use of Cardioplexol™), whose role was here to observe the administration procedure and if necessary, to intervene in order to prevent the occurrence of a procedure deviation. Only if the trainer was satisfied with the surgeon's performance was the latter allowed to further operate on his/her own using the Cardioplexol™ solution. If deemed necessary by the trainer, a third patient was scheduled and also operated in his presence.
- Part 2 (Analysis): Each surgeon's next consecutive 4 cases were performed without the presence of the coach. Data related to cardioplegia administration (volume and timing) were collected throughout the procedure by the perfusionist in charge, usually assisted by a second perfusionist, both specially trained to precisely record this information.

### Surgery procedure

The procedure was performed via total or partial sternotomy. The surgical approach was basically identical to that of standard practice in each center. The ascending aorta and the right atrium (or both vena cavae) were cannulated and a usual cardioplegia cannula was inserted into the ascending aorta, connected to a three-way stop-cock and de-aired. Extracorporeal circulation (ECC) was then started, and the flow was increased to 100% to completely unload the heart chambers. The ascending aorta was then clamped distally to the aortic root canula, and Cardioplexol™ solution was immediately administered. The operation then proceeded as usual.

### Investigational drug

Cardioplexol™ is a low volume cardioplegic solution. Its composition, preparation and administration were recently described (10). Briefly, the solution comes in a vial (containing 95 ml of a solution A) and a syringe (containing 5 ml of a solution B), both maintained in a fridge at 2–8°C prior to surgery. Approximately 30 min prior to administration, the content of the syringe is injected into the vial. The mixture of solutions A and B, forming the 100 ml ready-to-use Cardioplexol™ solution, is then distributed into two 50 ml syringes which are then to be injected by the surgeon him/ herself immediately after aortic cross-clamping.

### Study endpoints

The primary objective of the current study was to explore the effects of a training program on the rate of correct application of Cardioplexol™ intended to induce the cardiac arrest. For this purpose, the number of major deviations from the protocol of Cardioplexol™ administration (incorrect volume of initial dose, incorrect volume of second/third/fourth dose, incorrect duration of injection of initial dose, incorrect timing of application of initial dose, incorrect timing of application of second/third/fourth dose as detailed in Table 1) was set as primary endpoint.

**Table 1:** Description of primary and secondary endpoints.

Primary endpoint	
-Number of major deviations from the correct application of Cardioplexol™ as determined by the pre-specified training procedure: • initial dose - correct timing of application: max 60 s after aortic cross-clamping - correct duration of injection: max. 90 s - correct volume: 100–200 ml (in case of remaining activity, the surgeon may decide to administer a complement of 50–100 ml) • second dose: - correct timing of application: max. 60 min. after aortic cross-clamping - correct volume: 50–100 ml • Third dose: - correct timing of application: max. 90 min. after aortic cross-clamping - correct volume: 50–100 ml • fourth dose: - correct timing of application: max. 120 min. after aortic cross-clamping - correct volume: 50–100 ml	
Secondary endpoints	
1	Evolution of troponin T (TnT) values during the first 24 h following myocardial reperfusion
2	Evolution of creatinine kinase isoenzyme muscle-brain (CK-MB) values during the first 24 h following myocardial reperfusion
3	Time between the aortic cross-clamping and the complete cardiac arrest
4	Cumulative dose of catecholamines during aortic cross-clamping
5	Defibrillation rate after aorta unclamping and coronary reperfusion
6	Cumulative dose of catecholamines during the first 24 h following coronary reperfusion
7	Percentage of patients requiring the installation of an IABP during the first 24 h following coronary reperfusion or until ICU discharge (if discharge occurs before 24 h).
8	Duration of ICU stay
9	Mortality during the first 24 h following coronary reperfusion
Safety endpoints	
1	Serious and non-serious adverse events
2	Laboratory parameters

The secondary objective was to explore the effects of Cardioplexol™ on myocardial protection during the “ischemic” period to allow a rapid and complete reversibility of the cardiac arrest and the restoration of a normal cardiac contractile function following an intervention under the assistance of a heart-lung machine. Primary, Secondary and Safety endpoints are summarized in Table 1.

### Sample size calculation and statistical analysis

In the current study, it was expected that the training program would dramatically reduce the risk of incorrect administrations and that its number would be close to zero. However, even if no incorrect administration was to be observed, the true incidence would still have been greater than zero. This study was thus designed to show that the upper 95% confidence interval for the probability of incorrect administrations was below 0.03 under the assumption that no incorrect administration would be observed. It would thus require a sample size of 100 and the observation of no major deviation, to obtain an upper bound of 0.03 on the 95% confidence interval for the probability of major deviations.

The study analysis sets were defined as follows:

- Screening Set: All subjects who were screened (i.e., gave informed consent).
- Safety Set: All subjects who received at least one dose of the study medication.
- Training Set: All patients of the Safety Set who were included in the Training Part (Part 1).
- Analysis Set: All patients of the Safety Set who were included in the Analysis Part (Part 2).

The primary endpoint was assessed only for patients included in Part 2 (Analysis Set).

Descriptive statistics (number of patients, arithmetic mean, standard deviation, minimum, 1st quartile, median, 3rd quartile and maximum for continuous variables; number of patients, frequencies and percentages for categorical variables) are provided separately for patients included in part 1, in part 2, as well as for the entire collective.

## Results

### Patient disposition, demographic, pre-operative and surgical characteristics

**Figure 1:**
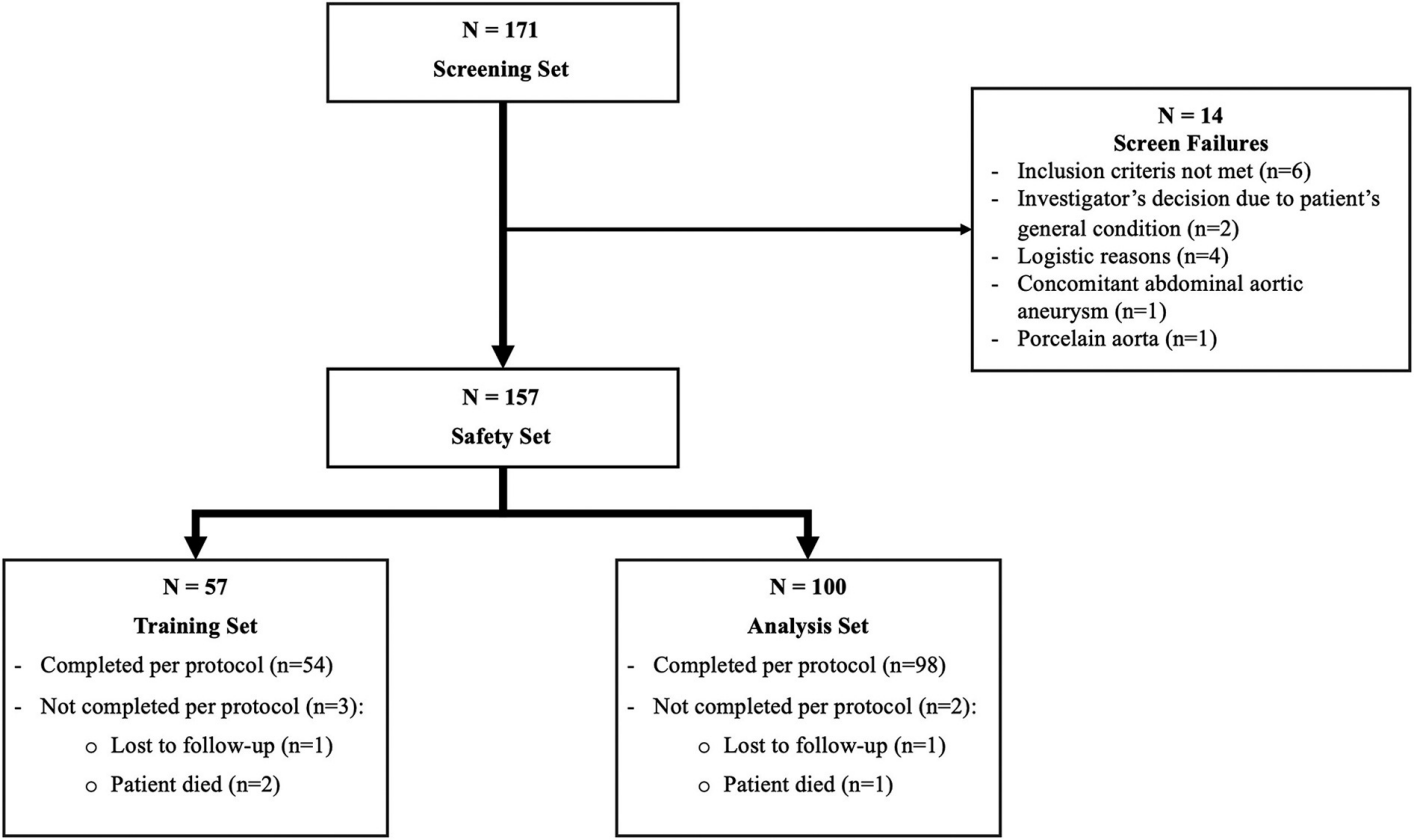
Patients disposition.

Patients disposition is shown in Figure 1. A total of 171 patients in Austria (106), Germany (34) and Switzerland (31) gave their informed consent to take part in the study. Fourteen of them were eventually not considered due to initially unanticipated anatomical, clinical or logistical reasons.

The Safety Set therefore included 157 patients. Surgeons operated 57 patients as part of their training program (part 1: Training Set), from which 54 completed the protocol, and 100 patients without a trainer (part 2: Analysis Set), from which 98 completed the protocol. Distribution between centers is shown in Supplementary Table S1.

The baseline profile of the 157 patients operated with Cardioplexol™ is shown in Table 2. Surgical parameters are shown in Table 3.

**Table 2:** Patients' demographic and pre-operative characteristics.

	Training Set *n* = 57	Analysis Set *n* = 100	Total *N* = 157
Demographics			
Age (years)	66.5 ± 8.0	66.5 ± 9.0	66.5 ± 8.6
Gender (male)	47 (82.5%)	76 (76.2%)	123 (78.3%)
BMI (kg/m^2^)	27.72 ± 4.0	27.9 ± 4.2	27.6 ± 4.1
Patient risk factors			
Smoker			
⚬ Current	13 (22.8%)	17 (17.0%)	30 (19.1%)
⚬ Former	16 (28.1%)	37 (37.0%)	53 (33.8%)
⚬ Never	25 (43.9%)	38 (38.0%)	63 (40.1%)
Diabetes (yes)	18 (31.6%)	27 (27.0%)	45 (28.7%)
Dyslipidemia (yes)	43 (75.4%)	81 (81.0%)	124 (79.0%)
Systemic hypertension (yes)	47 (82.5%)	87 (87.0%)	134 (85.4%)
Cardiovascular parameters			
NYHA class			
⚬ I	12 (21.1%)	18 (18.0%)	30 (19.1%)
⚬ II	21 (36.8%)	46 46.0%)	67 (42.7%)
⚬ III	16 (28.1%)	26 (26.0%)	42 (26.8%)
⚬ IV	2 (3.5%)	1 (1.0%)	3 (1.9%)
⚬ Unknown	6 (10.5%)	9 (9.0%)	15 (9.6%)
Coronary artery disease (yes)	49 (86.0%)	92 (92.0%)	141 (89.8%)
Cerebrovascular disease (yes)	7 (12.3%)	27 (27.0%)	34 (21.7%)
Normal coronaries (yes)	8 (14.0%)	9 (9.0%)	17 (10.8%)
LM stenosis > 50% (yes)	21 (36.8%)	40 (40.0%)	61 (38.9%)
Number of diseased vessels			
⚬ 1	2 (3.5%)	6 (6.0%)	8 (5.1%)
⚬ 2	11 (19.3%)	19 (19.0%)	30 (19.1%)
⚬ 3	34 (59.6%)	66 (66.0%)	100 (63.7%)
Left ventricular contractile function			
⚬ Good (>50% EF)	40 (77.2%)	77 (77.0%)	121 (77.1%)
⚬ Moderate (30–50% EF)	13 (22.8%)	23 (23.0%)	36 (22.9%)
Current angina (yes)	15 (26.3%)	27 (27.0%)	42 (26.8%)
History of myocardial infarction >7 days prior to surgery (yes)	17 (29.8%)	23 (23.0%)	40 (25.5%)
Arrhythmia (other than atrial fibrillation) (yes)	4 (7.0%)	5 (5.0%)	9 (5.7%)
Cardiac base rhythm is a sinus rhythm (yes)	53 (93.0%)	94 (94.0%)	147 (93.6%)

Results are expressed as mean ± SD or *n* (%). No relevant differences were observed between the training and analysis sets.

**Table 3:** Patients' operative characteristics.

	Training Set *n* = 57	Analysis Set *n* = 100	Total *N* = 157
CABG	46 (80.7%)	91 (91.0%)	137 (87.3%)
Aortic valve replacement	10 (17.5%)	10 (10.0%)	20 (12.7%)
Mitral valve repair/replacement	5 (8.8%)	2 (2.0%)	7 (4.5%)
Tricuspidal valve repair/replacement	3 (5.3%)	2 (2.0%)	5 (3.2%)
Aortic root repair	1 (1.8%)	2 (2.0%)	3 (1.9%)
ASD/PFO	1 (1.8%)	2 (2.0%)	3 (1.9%)
Septal myectomy	0	1 (1.0%)	1 (0.6%)
Cross-clamp time (minutes)	56.6 ± 19.3	57.7 ± 19.7	57.3 ± 19.5

Results are expressed as mean ± SD or *n* (%). No relevant differences were observed between the training and analysis sets.

### Training part

A total of 29 surgeons were recruited to participate to the study. All successfully completed the theoretical part of their training and were authorized to participate in Part 1. Twenty-eight surgeons completed the training part (Part 1) and successfully operated on two patients each. The trainer's assessment of the surgeons' performances during these 2 training procedures is shown in Supplementary Table S2. None of the surgeon required a third operation within this Training part. One surgeon was only temporarily available for the study and eventually operated, successfully, on only one training patient.

### Primary endpoint

The primary endpoint was assessed based on the Analysis set (Part 2) only. No major deviations from the correct application of Cardioplexol™ were observed: the volumes of the initial, second, third and fourth doses were correctly respected, as were the duration of the injection of the initial dose and the timing of the application of the initial, second, third and fourth doses (Table 4).

**Table 4:** Detailed analysis of primary endpoint components: number incorrect volume of initial dose, incorrect volume of second/third/fourth dose, incorrect duration of injection of initial dose, incorrect timing of application of initial dose, incorrect timing of application of second/third/fourth dose as detailed in Table 1.

	Training Set *n* = 57	Analysis Set^b^ *n* = 100	Total *N* = 157
Initial Dose	*n* = 57	*n* = 100	*n* = 157
Time to initial dose (sec)^a^	14.0 ± 12.8 (10, 0, 59)	12.0 ± 0.9 (10, 0, 60)	12.7 ± 11.6 (10, 0, 60)
Duration of initial dose injection (sec)	21.4 ± 12.3 (16, 4, 60)	20.4 ± 11.7 (19, 4, 69)	20.8 ± 11.9 (18, 4, 69)
Volume of initial dose (ml)	125.4 ± 39.1 (100, 100, 200)	124.0 ± 39.9 (100, 100, 300)	124.5 ± 39.5 (100, 100, 300)
Second dose	*n* = 28	*n* = 44	*n* = 72
Time to second dose (min)^a^	45.1 ± 8.7 (45.9, 17.3, 59.6)	47.7 ± 9.1 50.1, 15.7, 59.0)	46.7 ± 9.0 47.8, 15.7, 59.6)
Duration of second dose injection (sec)	11.1 ± 7.3 (8, 4, 28)	15.0 ± 10.5 (12, 2, 56)	13.5 ± 9.5 (11, 2, 56)
Volume of second dose (ml)	75.9 ± 25.0 (87.5, 50, 100)	83.1 ± 23.3 (100, 50, 100)	80.3 ± 24.1 (100, 50, 100)
Third dose	*n* = 2	*n* = 8	*n* = 10
Time to third dose (min)^a^	73.0 ± 6.4 (73.1, 68.5, 77.6)	73.9 ± 15.5 (78.5, 48.0, 89.1)	73.8 ± 13.8 (77.3, 48.0, 89.1)
Duration of third dose injection (sec)	9.5 ± 6.3 (10, 5, 14)	17.9 ± 18.8 (10, 5, 58)	16.2 ± 17.1 (10, 5, 58)
Volume of third dose (ml)	50.0 ± 0.0 (50, 50, 50)	69.6 ± 25.9 (50, 50, 100)	65.0 ± 24.1 (50, 50, 100)
Fourth dose	*n* = 0	*n* = 1	*n* = 1
Time to fourth dose (min)^a^	N.A.	117	117
Duration of fourth dose injection (sec)	N.A.	11	11
Volume of fourth dose (ml)	N.A.	50.0	50.0

Results are expressed as mea*n* ± SD and (median, min, max values). No significant differences were observed between the training and analysis sets (*p* > 0.05).

^a^ Time from aortic cross clamping until beginning of injection of initial, respectively second, third or fourth dose of cardioplegia.

^b^ Relevant for the evaluation of the primary endpoint.

### Secondary efficacy endpoints

Results of secondary endpoints are summarized in Table 5. Details regarding the profiles of TnT and CK-MB evolutions over the first 24 h post reperfusion are shown in Supplementary Table S3. Other relevant information includes the median duration of intubation of 7.4 h (min: 2.1–max: 192.4), and the median duration of hospitalization of 8 days (min: 3–max: 28).

**Table 5:** Results of secondary endpoints.

Secondary endpoint		Statistics	Training Set *n* = 57	Analysis Set *n* = 100	Total *N* = 157
1	Maximal value of TnT within the first 24 h ng/ml)^a^	Median IQR 95% CI	0.83 (0.47–1.27) 0.65–0.99	0.80 (0.50–1.31) 0.71–1.02	0.82 (0.49–1.30) 0.73–0.95
2	Maximal value of CK-MB within the first 24 h (U/L)^a^	Median IQR 95% CI	42.5 (32.0–60.0) 41.1–54.8	43.5 (31.0–60.2) 42.5–54.8	43.0 (32.0–60.0) 43.8–53.6
3	Time between aortic cross-clamping and complete cardiac arrest (sec)	Median range	10 (3–42)	12 (0–360)	10 (0–360)
4	Cumulative dose of catecholamines during aortic cross-clamping^b^	Median range	376 (6–3,029)	279 (10 – 36,780)	318 (6 – 36,780)
5	Defibrillation events after aorta unclamping and coronary reperfusion	Yes	1 (1.8%)	6 (6.0%)	7 (4.5%)
6	Cumulative dose of catecholamines during the first 24 h^b^	Median range	2,418 (30–223,780)	3,609 (8–500,000)	3,245 (8–500,000)
7	Use of IABP during the first 24 h	Yes	0	1 (1.0%)	1 (0.6%)
8	Duration of ICU stay (hours)	Median range	21.5 (7.0–262.0)	21.4 (1.2–184.0)	21.5 (1.2–262.0)
9	Death within the first 24 h	Yes	0	0	0

^a^ Median, interquartile range (IQR) and CI calculated based on back transformed (anti log) values.

^b^ Calculated according to Cruz et al, JAMA 2009 (16).

### Secondary safety endpoints

The list of adverse events is summarized in Supplementary Table S4. Their distribution according to severity grade and relationship to the study drug (causality) is summarized in Supplementary Table S5. Two patients died within 30 days of surgery (1.3%) due respectively to severe bilateral pneumonia and neurological complications. One additional patient (0.6%) died of severe respiratory decompensation at 32 days post-surgery. None of the three fatal evolutions was related to the cardioplegia solution used.

## Discussion

Myocardial protection is crucial for the overall quality of a cardiac surgery procedure. The choice but also the correct administration of cardioplegia solution, may play a decisive role (11, 12). Cardioplexol™ solution has recently been the subject of a pivotal Phase 3 clinical trial, demonstrating its efficacy and safety (10). However, a few numbers of deviations from the drug administration protocol were observed, suggesting a training program could be beneficial. The aim of the current study was therefore to test the hypothesis that a specific, monitored training program, for surgeons without previous experience with Cardioplexol™, could improve the technique and reliability of administering this cardioplegia solution, and thus prevent or at least critically reduce the risk of administration error, which in turn might improve the overall benefit of myocardial protection.

The study protocol included for each candidate a learning phase (part 1) and an evaluation phase during which the effect of this learning was assessed (part 2). The results were convincing. First, we observed that each candidate successfully completed the theoretical part of the training course and adequately answered the series of questions related to Cardioplexol™ administration. Each candidate was thus authorized to start operating with Cardioplexol™. Here as well, each candidate successfully operated his/her two first patients under the attentive supervision of the trainer who never had to take any action with regard to Cardioplexol™ administration. The trainer's assessment of these 2 training procedures therefore confirmed that the surgeon's handling of the Cardioplexol™ solution was fully appropriate. Under these circumstances, each surgeon was thus cleared to perform further procedures with Cardioplexol™ and without any supervision.

For these additional procedures, none of the evaluated surgeons committed any application error. In other words, each aspect considered for the assessment of the quality of Cardioplexol™ administration was fully respected. It can be therefore concluded that the adoption of a structured, monitored training program efficiently prevents, or at least significantly reduces, the risk of cardioplegia administration errors.

Aside from the question regarding the training, the present study also allows to address other criticisms made about the first pivotal study. Indeed, the current study has been designed in such a way as to enable direct comparison of its secondary endpoints with those primary and secondary endpoints assessed in the first pivotal study. For instance, one of the limitations of this first study was that it presented results from only one single center and, as such, may not be representative of a more general situation. With the present study, results from 7 new centers, 29 new surgeons and 2 additional European countries have been added. And these results, all positive, now confirm those presented in the pivotal study (10), and notably the favorable post-operative profiles of myocardial enzyme elevation. For instance, max TnT value within 24 h post-reperfusion is now 0.82 ng/ml (95% CI: 0.73–0.95) and was 0.84 ng/ml (075–0.95) in the pivotal study. Max CK-MB value is now 43.0 U/L (95% CI: 43.8–53.6) vs. 56.7 U/L (95% CI: 51.0–63.0) in the previous study. Also, the results of other secondary endpoints appear to be very similar or even better than those observed in the pivotal study. For example, the cumulative inotropic support provided during the procedure and especially during the first 24 h after myocardial reperfusion appears significantly lower in the current new study than in the initial study. Similarly, the rate of cardioversion at the end of the procedure was extremely low (<5% vs. 12.6% in the pivotal study), and the average length of stay in intensive care was less than 24 h (vs. 37.8 in the pivotal study).

Training in the proper use of a new technique, such as robotic surgery or new surgical devices, is now common practice (13–15). In fact, many of these new approaches may be highly specific and complex that an error could have dramatic, consequences. The aim of such a training is therefore to improve both the efficacy and safety of these techniques or devices. In the case of drug administration, such a program is exceptional and generally concerns the use of a device linked to its administration, rather than to the administration of the drug itself (16). To our knowledge, the program presented in our current study may be the first one dedicated to the administration of a new drug and the first case where it was an integral part of its safety control. Our current results demonstrate that even for an initially considered straightforward application of a new drug, the overall efficiency and safety of that drug can be critically improved by a dedicated administration training program. Together with the earlier pivotal study (10), the results now confirm that both the benefit of the new Cardioplexol™ solution, combined with proper administration technique, provide excellent results in terms of efficiency and safety.

## Limitations

The present study was thought and designed to address the limitations identified during the European approval process of Cardioplexol™. The study involved several new surgeons, centers and two additional countries. Although the results are clearly positive and satisfied the EU authorities, as with other new devices or drugs, further studies will be needed to extend the validation of the safety and efficacy of the Cardioplexol™ solution, especially in sub-groups of patients.

## Conclusion

Cardiac surgeons without previous experience with the use of Cardioplexol™ solution clearly benefit from a structured and supervised training program. Such a training program appear to contribute to improving the efficacy and safety of a new cardioplegia solution such as the Cardioplexol™ solution.

The data presented in the current report, together with that featured in the pivotal study (10), formed an instrumental part of the Cardioplexol™ registration application. Marketing authorization was eventually granted in Switzerland in September 2023 and in 10 European countries in April 2024. Further studies as well as a registry of newly trained surgeons and patients operated on with Cardioplexol™ are currently being planned.

## Data Availability

The datasets presented in this study can be found in online repositories. The names of the repository/repositories and accession number(s) can be found in the article/Supplementary Material.
