# Supplementary material for: A multi-center, open label, single group, observational clinical trial to investigate the effects of training on the administration of Cardioplexol™
Source: Front Cardiovasc Med. 2025 May 26;12:1588088. doi: 10.3389/fcvm.2025.1588088 (PMC12146177; doi:10.3389/fcvm.2025.1588088)
Supplement: Supplementary file 1 [file Datasheet1.pdf]

A Multi-Center, Open Label, Single Group, Observational Clinical Trial to Investigate the Effects of Training on the Administration of Cardioplexol™

Supplementary Material

Supplementary Material - 1

Training program

The purpose of this program is to train cardiac surgeons and cardiotechnicians who are unexperienced with the use of Cardioplexol™ and who are willing to operate with this cardioplegic solution. Ultimately, the purpose of this training is to increase the efficacy of the CardioplexolÔ administration while reducing the risk of false manipulations.

Training objectives

1. Learn how to correctly prepare the Cardioplexol™ solution

- This part concerns mostly but not exclusively the cardiotechnicians. Storage aspects will be discussed as well as the correct methods of preparing the solution itself until it is delivered to the scrub nurse.

2. Learn how to correctly administer the Cardioplexol™ solution

- This part concerns mostly but not exclusively the surgeons. Surgeons will indeed deliver the solution themselves and this directly into the aortic root. Besides the methods of Cardioplexol™ injection, other critical aspects will be discussed, including especially the correct volume to be administered and the correct timing of administration of the initial and repetition doses.

Training principles

The training program is managed by dedicated coaches with large clinical experience with Cardioplexol™. It is designed to teach cardiac surgeons and cardiotechnicians the correct methods of preparation and administration of the Cardioplexol™ cardioplegic solution. It includes three consecutive parts:

- - ***a theoretical part*** during which the various aspects of Cardioplexol™ preparation and administration will be presented and discussed
- - ***a practical part*** during which the Cardioplexol™ preparation and administration will be exercised
- - ***a clinical part*** by which the first 2 or 3 Cardioplexol™ patients will be operated in the presence of a coach

The theoretical and practical parts will be given in one block of approximately 2-3 hours and will be organized in small groups of participants. Material in electronic and paper form will be distributed in advance to each candidate who will be invited to read the brochure in advance in

order to get familiar with the Cardioplexol™ solution, its administration principles and particularities, as well as the latest clinical results.

Training evaluation

At the end of the first 2 parts, candidates will be asked to answer in written and questionnaire individually, to ensure sure that every critical aspect of the preparation and administration of Cardioplexol™ solution is fully assimilated. In the event of err, the particular points will be discussed individually with the concerned participant. Candidates will only be allowed to participate in the third, clinical part of their training if they have successfully completed the first theoretical aspects of the training, including the questionnaire, and if the coach is satisfied with their performance.

The first two Cardioplexol™ surgeries will be performed in the presence of the trainer, who will monitor and, if necessary, correct the administration procedure. If satisfied with the surgeon's performance, he will authorize him/her to perform the subsequent operations on his own. If not, a third patient will be scheduled in the presence of the trainer.

Supplementary Table 1

Distribution of patients between the seven study centers

	**Training Set**<br>**n = 57**	**Analysis Set**<br>**n = 100**	**Total**<br>**N = 157**
Clinic Floridsdorf Vienna	7	12	19
University Hospital St.Pölten	7	15	22
University Hospital Salzburg	12	24	36
University Hospital Innsbruck	8	13	21
University Hospital Frankfurt	7	8	15
German Heart Center Berlin	6	8	14
University Hospital Zürich	10	20	30

Supplementary Table 2

Trainer’s assessment of surgeons’ performance during their two initial training procedures with Cardioplexol™

	**First patient**<br>**N = 29**	**Second patient**<br>**N = 28**
	**Median (min, max)**	
Appears confident	10 (8, 10)	10 (8, 10)
Follows strictly the instruction	10 (8, 10)	10 (9, 10)
Communicates efficiently with the cardiotechnician	10 (8, 10)	10 (10, 10)
Reacts correctly to the information	10 (10, 10)	10 (10, 10)
Anticipates correctly	10 (10, 10)	10 (9, 10)
Manipulates the solution correctly	10 (8, 10)	10 (9, 10)

Supplementary Table 3

Details of TnT and CK-MB values at 3, 6, 12 and 24 hours post-myocardial reperfusion

	**Training Set N=57**	**Analysis Set N=100**	**Total N=157**
	**Median (min, max)**		
**Troponin T values (ng/mL)**			
3 hours	0.61 (0.10, 3.83)	0.58 (0.06, 24.84)	0.58 (0.06, 24.84)
6 hours	0.71 (0.16, 7.08)	0.75 (0.11, 28.88)	0.72 (0.11, 28.88)
12 hours	0.46 (0.13, 12.38)	0.51 (0.07, 18.59)	0.51 (0.07, 18.59)
24 hours	0.30 (0.08, 9.74)	0.35 (0.05, 20.91)	0.34 (0.05, 20.91)
Max. TnT value	0.83 (0.16, 12.38)	0.81 (0.11, 28.88)	0.82 (0.11, 28.88)
**CK-MB (U/l)**			
3 hours	39 (20, 181)	40 (23, 466)	40 (20, 466)
6 hours	35 (18, 365)	37 (13, 413)	36 (13, 413)
12 hours	35 (16, 501)	33 (15, 464)	34 (15, 501)
24 hours	32 (12, 308)	28 (9, 379)	29 (9, 379)
Max. TnT value	43 (20, 501)	44 (16, 466)	43 (16, 501)

Supplementary Table 4

Adverse events coded according to the MedDRA preferred terms

	**Training set (TS)** **N = 57**	**Analysis set (AS)** **N = 100**	**Total (SS)** **N = 157**
		**no. of patients (%)**	
**Blood and lymphatic system disorders**			
Anaemia	43 (75.4%)	76 (76.0%)	119 (75.8 %)
**Cardiac disorders**			
Atrial fibrillation	15 (26.3%)	28 (28.0%)	43 (27.4%)
Pericardial effusion	3 (5.3%)	6 (6.0%)	9 (5.7%)
**Gastrointestinal disorders**			
Nausea	7 (12.3%)	8 (8.0%)	15 (9.6%)
Constipation	1 (1.8%)	6 (6.0%)	7 (4.5%)
**General disorders and administration site conditions**			
Pain	10 (17.5%)	20 (20.0%)	30 (19.1%)
Pyrexia	3 (5.3%)	8 (8.0%)	11 (7.0%)
Oedema peripheral	0	6 (6.0%)	6 (3.8%)
**Infections and infestations**			
Pneumonia	4 (7.0%)	3 (3.0%)	7 (4.5%)
**Injury, poisoning and procedural complications**			
Anaemia postoperative	10 (17.5%)	20 (20.0%)	30 (19.1%)
Wound dehiscence	3 (5.3%)	4 (4.0%)	7 (4.5%)
Postoperative delirium	3 (5.3%)	3 (3.0%)	6 (3.8%)
**Investigations**			
C-reactive protein increased	4 (7.0%)	5 (5.0%)	9 (5.7%)
Inflammatory marker increased	2 (3.5%)	5 (5.0%)	7 (4.5%)
Myocardial necrosis factor increased	1 (1.8%)	6 (6.0%)	7 (4.5%)
**Metabolism and nutrition disorder**			
Hyperglycaemia	1 (1.8%)	5 (6.0%)	6 (3.8%)
**Psychiatric disorders**			
Delirium	3 (5.3%)	11 (11.0%)	14 (8.9%)
Sleep disorder	4 (7.0%)	8 (8.0%)	12 (7.6%)
**Renal and urinary disorders**			
Acute kidney injury	4 (7.0%	8 (8.0%)	12 (7.6%)

Supplementary Table 5

Distribution of adverse events according to their severity grade, outcome and causality

	**Training set (TS) N=57**	**Analysis set (AS) N=100**	**Total (SS) N=157**
	**no. of AEs (%)**		
**Any AE**	218 (100.0%)	460 (100.0%)	678 (100 %)
**AE Grade**			
1: mild	111 (50.9%)	213 (46.3%)	324 (47.8%)
2: moderate	85 (39.0%)	207 (45.0%)	292 (43.1%)
3: severe	22 (10.1%)	40 (8.7%)	62 (9.1%)
**SAE**	51 (23.4%)	102 (22.2%)	153 (22.6%)
**Outcome**			
Recovered/ resolved	162 (74.3%)	332 (72.2%)	494 (72.9%)
Recovering/resolving	33 (15.1%)	64 (13.9%)	97 (14.3%)
Not recovered/ not resolved	15 (6.9%)	34 (7.4%)	49 (7.2%)
Recovered/ recovered with sequelae	2 (0.9%)	23 (5.0%)	25 (3.7%)
Fatal	2 (0.9%)	1 (0.2%)	3 (0.4%)
Unknown	4 (1.8%)	6 (1.3%)	10 (1.5%)
**Causality/relationship according to safety office**			
certain	0	0	0
probable	0	0	0
possible	3 (1.4%)	5 (1.1%)	8 (1.2 %)
unlikely	2 (0.9%)	6 (1.3%)	8 (1.2%)
not related	213 (97.7%)	449 (97.6%)	662 (97.6%)
not assessable	0	0	0
unknown	0	0	0
**Causality/relationship according to study centers**			
certain	0	0	0
probable	0	0	0
possible	6 (2.8%)	11 (2.4%)	17 (2.5 %)
unlikely	2 (0.9%)	9 (2.0%)	11 (1.6%)
not related	209 (95.9%)	440 (95.7%)	649 (95.7%)
not assessable	0	0	0
unknown	1 (0.5%)	0	1 (0.1%)
